# *Drosophila* renal stem cells enhance fitness by delayed remodeling of adult Malpighian tubules

**DOI:** 10.1126/sciadv.abn7436

**Published:** 2022-05-20

**Authors:** Chenhui Wang, Allan C. Spradling

**Affiliations:** Department of Embryology, Carnegie Institution for Science, Howard Hughes Medical Institute, Baltimore, MD 21218 USA.

## Abstract

*Drosophila* renal stem cells (RSCs) contradict the common expectation that stem cells maintain tissue homeostasis. RSCs are abundant, quiescent, and confined to the peri-ureter region of the kidney-like Malpighian tubules (MTs). Although derived during pupation-like intestinal stem cells, RSCs initially remodel the larval MTs only near the intestinal junction. However, following adult injury to the ureter by xanthine stones, RSCs remodel the damaged region in a similar manner. Thus, RSCs represent stem cells encoding a developmental redesign. The remodeled tubules have a larger luminal diameter and shorter brush border, changes linked to enhanced stone resistance. However, RSC-mediated modifications also raise salt sensitivity and reduce fecundity. Our results suggest that RSCs arose by arresting developmental progenitors to preserve larval physiology until a time in adulthood when it becomes advantageous to complete the development by RSC activation.

## Teaser

Activated *Drosophila* renal stem cells rebuild the adult Malphigian tubules using a less-efficient but more stone-resistant design.

## INTRODUCTION

Many tissues such as skin and intestinal epithelium have adult stem cells, which sustain homeostatic tissue function by regulated self-renewal and daughter cell differentiation. It is commonly assumed that all adult stem cells act to maintain the composition, morphology, and function of their tissue in the face of fluctuating environmental conditions, stress, and tissue damage (*1*–*3*). Here, we show that the unusual properties of the renal stem cells (RSCs) found within the *Drosophila* adult Malpighian tubule (MT) suggest that they represent a class of stem cells with an alternative function—tissue remodeling.

**Fig. 1.**
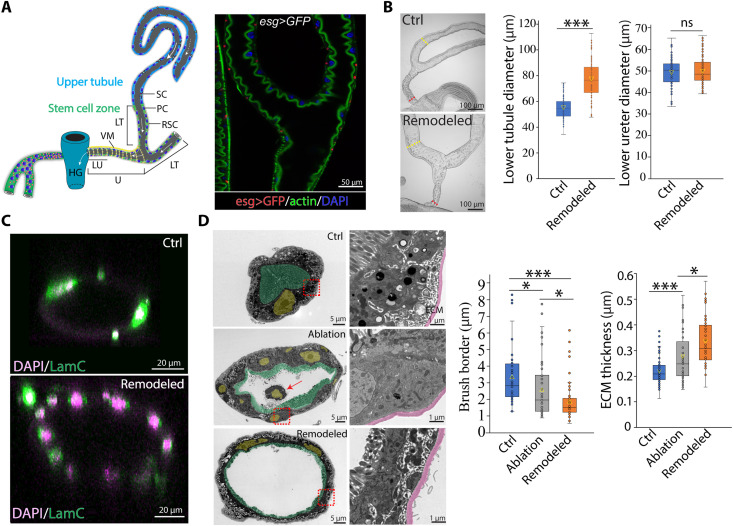
Morphological remodeling of adult MTs. (**A**) Left: Sagittal cross-sectional diagram of adult *Drosophila* MTs. Right: Immunofluorescence (IF) of ureter and lower tubules of *esg-Gal4>UAS-GFP* MT stained with phalloidin. PC, principal cell; VM, visceral muscle; SC, stellate cell; LT, lower tubule; U, ureter; LU, lower ureter; HG, hindgut. White arrows denote the direction of fluid flow along the tubules toward hindgut. The solid arrows denote the flow direction of fluid that is generated in the main segment, whereas the dashed arrow denotes reflux from the main segment into the initial/transitional segments (*6*). (**B**) RSC-mediated repair of the SCZ increases lower tubule diameter after 21 days at 18°C following genetic ablation of PCs at 29°C. Ctrl: *C507-Gal4^ts^/+*; remodeled: *C507-Gal4^ts^>UAS-rpr+hid*. Quantitation is at the right. (**C**) IF of control (Ctrl) lower tubule and remodeled (Remodeled) lower tubule. LamC, lamin C, a component of nuclear lamina. DAPI, 4′,6-diamidino-2-phenylindole. (**D**) Pseudo-colored transmission electron microscopy image showing thickened extracellular matrix (ECM; magenta) and shortened brush borders (green). PC nuclei (yellow). The red arrow denotes a dying cell in the lumen. Quantitation is at right. For the box and whisker plots, the yellow triangle indicates the mean value, whereas the line inside the box indicates median value. The two lines constitute the top and bottom of the box are the 25th and 75th percentiles, respectively. Two-tailed Student’s *t* test; ***P < 0.001; ns, not significant (*P* > 0.05). Scale bars are as indicated.

*Drosophila* MTs are functionally analogous to mammalian kidneys but with much simpler tissue structure (*4*). The MTs have little cell turnover under normal physiological condition but are vulnerable to damage under stress conditions (*5*). RSCs are only found within the peri-ureter region known as the stem cell zone (SCZ) of adult MTs (Fig. 1A), where they comprise 66% of tissue cells but usually remain quiescent (*5*). The SCZ corresponds to the ureter and lower tubules, which reabsorb 30% of fluid secreted by the upper tubules (*6*). The ureter is surrounded by visceral muscles that control fluid flow in the ureter via peristalsis (Fig. 1A) (*7*).

RSCs share a common origin with *Drosophila* intestinal stem cells (*8*). Both derive from the pool of adult midgut progenitors, which extensively remodel the larval midgut yet leave the larval MTs almost intact except the lower ureter region where large larval principal cells (PCs) are replaced with smaller PCs (*9*). Adult RSCs barely replenish any tubule cells under normal conditions. However, RSCs can be activated when renal stones damage PCs located up to about 10 cell diameters away. Instead of homeostatically replacing the damaged cells, RSC-derived replacement PCs are much smaller than preexisting PCs but are more abundant, similar to PCs in the lower ureter region (*5*).

## RESULTS

### *Drosophila* RSCs remodel adult MTs upon injury

We genetically ablated adult PCs to more fully understand the nature of RSC-mediated lower tubule repair (see Materials and Methods). While RSC-generated PCs are much smaller than preexisting PCs, the overall DNA content of the tissue is largely restored (fig. S1). However, we found that the diameter of lower tubules also increased significantly from 54.4 ± 8.2 μm (means ± SD; *n* = 107 tubules) before repair to 77.0 ± 14.3 μm following RSC-mediated remodeling (*P* = 1.79 × 10^−29^; *n* = 80 tubules; Fig. 1B and fig. S2). In contrast, the diameter of the lower ureter, which undergoes RSC-mediated remodeling during pupal development, remained unchanged between control animals (49.5 ± 6.4 μm; *n* = 75 ureters) and animals with remodeled MTs (50.0 ± 6.7 μm; *n* = 64 ureters) (Fig. 1B). Despite their increased diameter, remodeled MTs remained a monolayered epithelium, albeit one containing more cells (Fig. 1C). The brush border of replacement PCs is significantly shorter than that of preexisting PCs (1.60 ± 0.75 μm versus 3.37 ± 1.88 μm; *P* = 2.41 × 10^−6^; *n* = 8), whereas the extracellular matrix (ECM) thickness of the lower tubules substantially increases following repair (0.36 ± 0.09 μm versus 0.22 ± 0.05 μm; *P* = 1.02 × 10^−10^; *n* = 10) (Fig. 1D).

Because septate junctions (SJs) and adherens junction (AJ) are crucial for epithelial barrier function and cell adhesion in invertebrates, we examined the expression and localization of the conserved SJ protein Coracle and the AJ core component Armadillo in lower tubules following regeneration (*10*, *11*). Coracle is localized to the apicolateral SJ of the preexisting PCs, above the AJ domain to which Armadillo is localized, indicating that the SJs form above the AJs in these ectodermally derived PCs (fig. S3, A and B). Following remodeling, localization of Coracle and Armadillo in replacement PCs remains the same (fig. S3, A and B). These results suggest that RSC-mediated remodeling does not affect the proper localization of SJs and AJs (fig. S3C).

### Xanthine stones are found predominantly in the SCZ

**Fig. 2.**
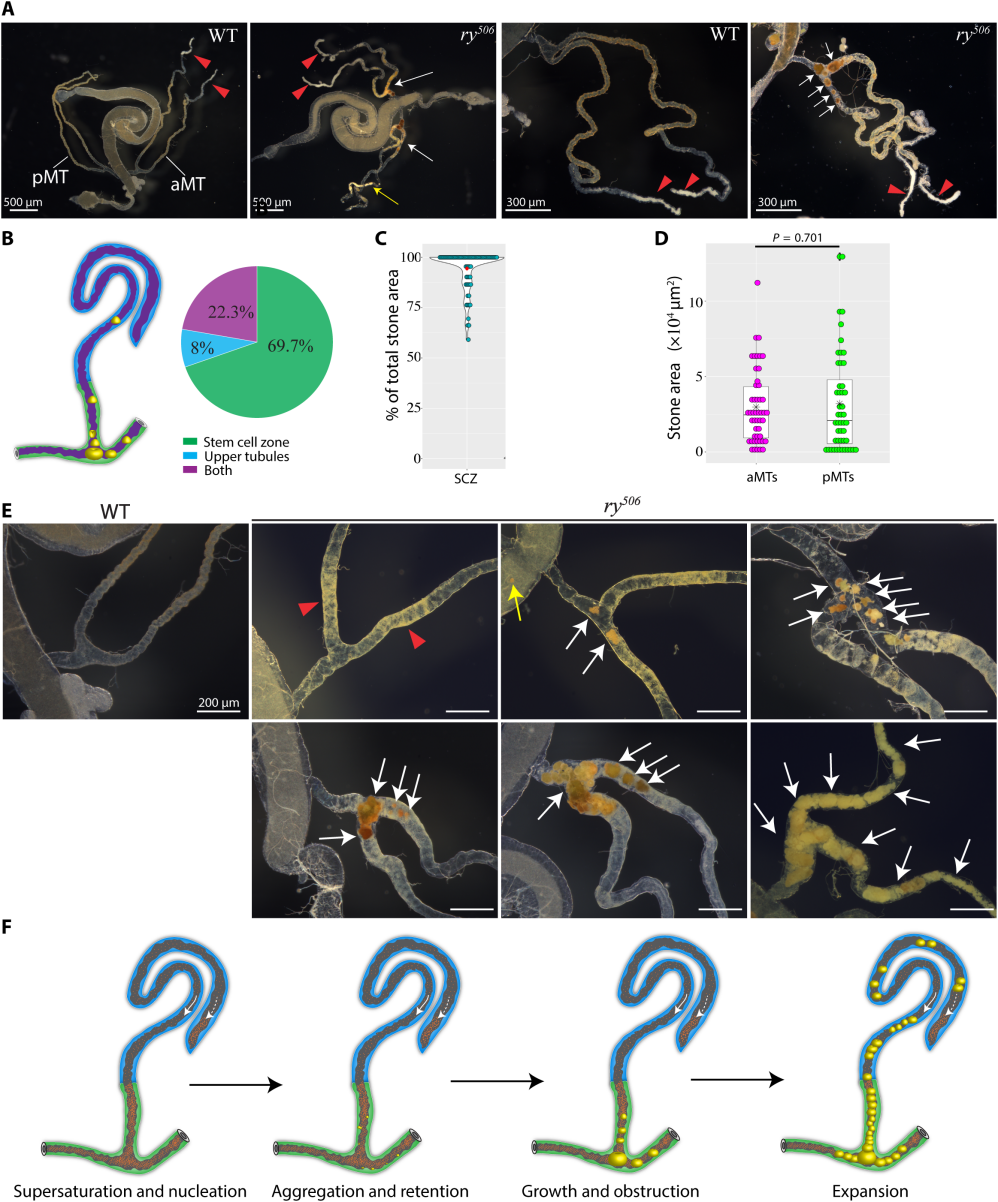
Xanthine stones are found predominantly in the lower MTs. (**A**) Preferential accumulation of xanthine stones (white arrows) in the ureter and lower tubules of *ry* mutants or wild-type (WT) controls. Xanthine stones in upper tubule (yellow arrow). Red triangles denote calcium calculi in anterior MT pairs (aMTs). Posterior MT pair (pMT). (**B**) Stone distribution in MTs (3- to 7-day-old well-fed *ry* mutants). (**C**) Percentage of stone mass (area) in the SCZ versus upper tubules. (**D**) Stone mass (area) distributions in aMTs and pMTs. (**E**) Xanthine stone formation (white arrows) in *ry* MTs. Red triangles denote lower tubules carrying tiny yellow particles. Stones are indicated in the hindgut (yellow arrow) and lower tubules (white arrows). Left: Control MT (WT). (**F**) Model of progressive xanthine stone formation in *ry* MTs. Scale bars, 200 μm or as indicated.

Kidney stones are a common renal disease in humans (*12*) that also affect insects such as *Drosophila* (*13*). The process of stone formation is readily studied using the *rosy* (*ry*) gene, which encodes xanthine dehydrogenase (XDH), as *ry* mutants develop xanthine-rich stones in their MTs at high frequency (*5*, *14*). We showed previously that xanthine stones elicit damage that triggers RSC-mediated repair in the SCZ (*5*). To further understand how xanthine stones form in *Drosophila* MTs, we first examined the distribution of stones in 3- to 7-day-old *ry* mutant animals that had been well fed after eclosion. Among *ry* mutant MTs bearing xanthine stones (*n* = 211), 69.7% had visible stones exclusively in the SCZ and 22.3% had discernible stones in both the SCZ and the upper tubules, whereas only 8% exclusively had stones in the upper tubules (Fig. 2, A and B). In addition, the vast majority of xanthine stone mass (94.7%; *n* = 72) was found in the SCZ, as assessed by area measurement (Fig. 2C). Therefore, RSCs are found in the MT region that is substantially more prone to acquire xanthine stones than other regions. This conclusion applied to both the anterior and posterior MT pairs, which differ in size and function. No difference in the average load of stones was found (Fig. 2D). Stone formation in anterior versus posterior tubules appeared to initiate independently, as little correlation in stone size was seen (fig. S4, A to D).

Studying the early stages of stone formation revealed an additional connection between RSCs and stones. The lower tubules of *ry* mutants were more yellow in color compared to other MT regions or to wild-type MTs, and small yellow particles could be seen to accumulate in the lower tubules before the appearance of stones (Fig. 2E). The lower tubule reabsorbs about 30% of fluid secreted by the upper tubules (*6*). Thus, the xanthine concentration in the lower tubules is more likely to become supersaturated than in the upper tubules, promoting stone nucleation (*15*). After nucleation, stones appeared sporadically in the SCZ but were often found in the hindgut as well, indicating that small stones can be excreted into the hindgut (Fig. 2E). The dynamics of retention in the MTs and washout via the hindgut likely mediate stone progression, which can vary between tubules in the same animal (fig. S4, A and B). Once retained in MTs, stones gradually grow in size and aggregate with each other to form larger stones that lead to obstruction and RSC activation (Fig. 2, E and F).

We previously found that RSCs enhance survival following allopurinol treatment, which directly inhibits XDH (*5*). To investigate whether RSCs are evolutionarily conserved, we examined the MTs from adult *Drosophila pseudoobscura*, which is estimated to have diverged from *Drosophila melanogaster* about 33 million years ago (*16*). We found a population of diploid cells strongly resembling RSCs in their ureters and lower tubules (fig. S5A). Moreover, allopurinol-induced stones preferentially start to accumulate in this MT region in *D. pseudoobscura*. RSCs can also repair the damage caused by stones in a similar manner as in *D. melanogaster*: Preexisting PCs are replaced by much smaller PCs in stone-damaged tubules (fig. S5, B and C). In addition, the lower tubule diameter is significantly increased in *D. pseudoobscura* bearing allopurinol-induced stones (fig. S5D).

We carried out similar studies of a more distantly related fly, the lower dipteran *Sciara coprophila*, which diverged from *D. melanogaster* early in dipteran evolution about 250 million years ago. In *Sciara*, the tubular epithelium only contains polyploid PCs in the ureter and lower tubules, suggesting the absence of RSCs (fig. S5E). Adult MT remodeling may have evolved after the divergence of *Sciara* and *Drosophila*, or it may have been lost in *S. coprophila* because adults of this species are short-lived compared to *Drosophila*.

### Remodeling enhances resistance to xanthine stones

**Fig. 3.**
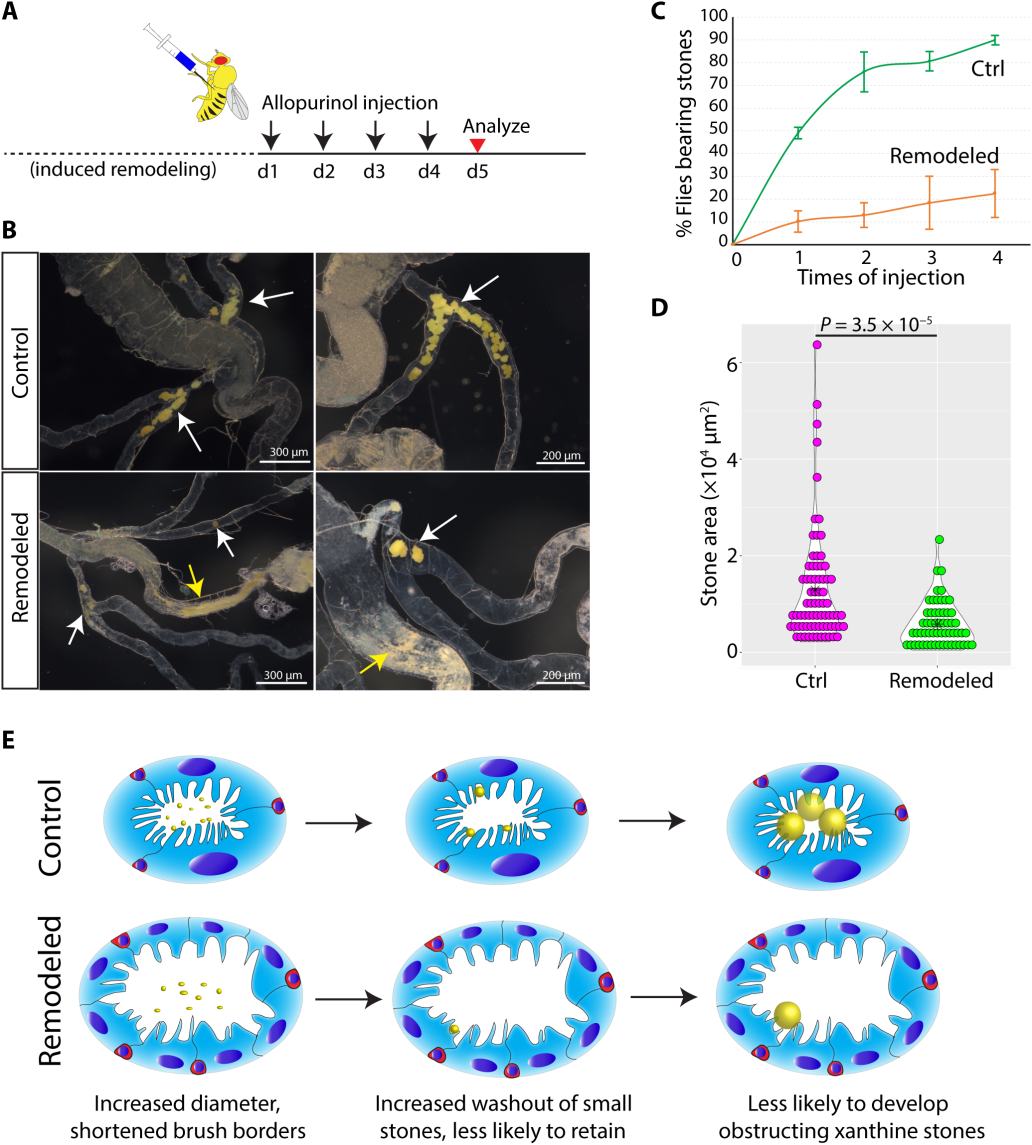
Remodeled MTs are stone-resistant. (**A**) Experimental scheme for xanthine stone induction by allopurinol injection following 14 days of recovery after induced remodeling (see Materials and Methods). d, day. (**B**) Xanthine stones (arrows) in control MTs (top) and remodeled MTs (bottom). Stones in SCZ (white arrows) and stones in hindgut (yellow arrows). (**C**) Control (Ctrl) flies are more susceptible to induced stones than flies with remodeled MTs (Remodeled). Error bars denote SD. (**D**) Stone size (area) distributions in MTs from control (Ctrl) and in animals with remodeled MTs (Remodeled) after 4 days of allopurinol injection. (**E**) Model for the enhanced stone resistance following RSC-mediated remodeling of adult MTs, showing increased luminal diameter and shortened brush border of tubules within the SCZ, which increases stone washout into the hindgut. Blue ovals denote polyploid PC nuclei, red cells denote RSCs, and yellow spheres denote stones. Scale bars are as indicated.

MT remodeling by RSCs to increase tubule diameter and shorten brush border length may enhance stone resistance. To probe this possibility, we developed a scheme to rapidly induce xanthine stones by injecting flies twice with allopurinol spaced by 24 hours (Fig. 3A). As we observed in *ry* mutants, stones induced by allopurinol injection preferentially appeared in the lower tubules. After allopurinol injection on four consecutive days, 89.9 ± 2.15% of control animals (*n* = 90) carried discernible stones in at least one of four MTs (Fig. 3, B and C). In contrast, only 22.4 ± 10.5% of flies (*n* = 78) with remodeled MTs bore visible stones (Fig. 3, B and C), a highly significant difference (*P* < 0.001). In addition, the total stone area in affected animals was significantly decreased in remodeled compared to control MTs (5840 ± 4700 μm^2^ versus 12,900 ± 11,800 μm^2^; *P* = 3.5 × 10^−5^) (Fig. 3D). Stones were more frequently observed in the hindguts of animals with remodeled MTs compared to control animals (Fig. 3B), consistent with increased expulsion of stones in remodeled MTs. A higher rate of loss likely contributes to the lower stone levels in remodeled MTs. Together, our data show that MT remodeling, including changes in diameter and brush border length, reduces the incidence of obstructing stones.

### Remodeling enhances salt sensitivity and reduces fertility

**Fig. 4.**
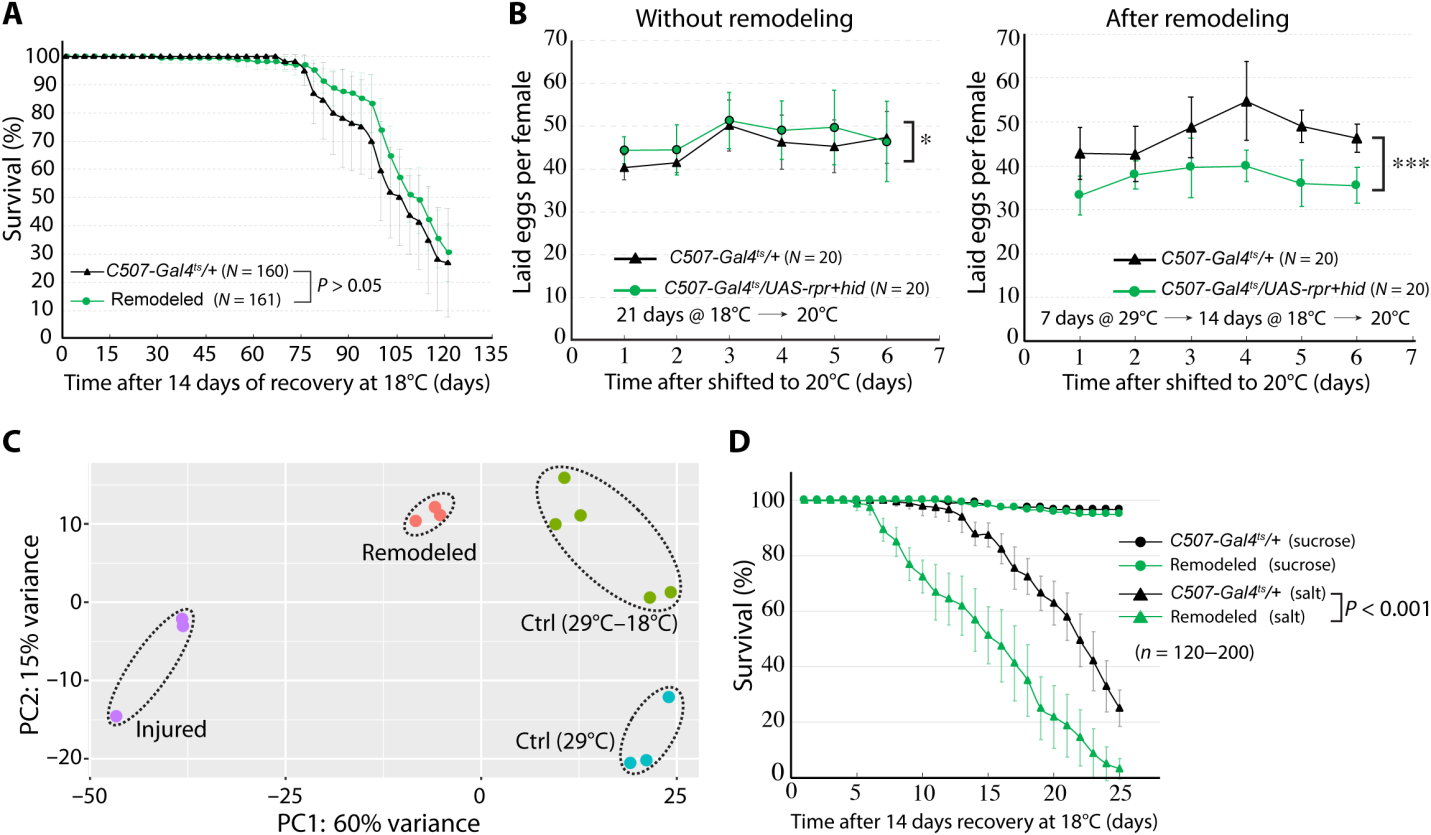
Remodeling of adult MTs compromises fecundity and high salt tolerance. (**A**) Survival curves (days) of controls (purple) and animals with remodeled MTs (green) at 18°C. (**B**) Fecundity (eggs laid per day per female) of control (*C507-Gal4^ts^/+*) or MT-remodeling competent (*C507-Gal4^ts^>UAS-rpr+hid*) females at 20°C. Left: Without remodeling (no heat treatment) before egg laying assay. Right: With MT remodeling (29°C for 7 days and 18°C for 14 days) before egg laying assay. (**C**) Principal components analysis plot of SCZ transcriptomes from Ctrl (*C507-Gal4^ts^/+*) females either at (29°C for 7 days; “29°C”) or at (29°C for 7 days and 18°C for 21 days; “29°C–18°C”); injured and remodeled (*C507-Gal4^ts^>UAS-rpr+hid*) females either at (29°C for 7 days; “Injured”) or (29°C for 7 days and 18°C for 21 days; “Remodeled”). Dots within dashed ellipses denote biological replicates. (**D**) Survival curves of flies with control (c507-Gal^ts^/+) or remodeled (Remodeled) MTs fed on 5% sucrose with or without 250 mM NaCl (“salt”). Error bars indicate SD. *P < 0.05 and ***P < 0.001; one-way repeated measures analysis of variance (ANOVA).

Because MT remodeling enhances stone resistance but does not take place throughout the MT as expected during pupal development, we looked for possible tradeoffs in fitness. We first examined whether MT remodeling affects the life span. Flies with remodeled MTs live as long and possibly longer than control animals fed on regular food at 18°C (Fig. 4A). We next examined whether adult MT remodeling affects the fecundity. Eggs laid per female following induced remodeling of MTs were measured for six consecutive days and compared to control. From the fourth day on, female flies with remodeled MTs produced 23 to 27% fewer eggs per day (*P* < 0.05; *n* = 20 flies) (Fig. 4B). Decreased fecundity was not due to differences in the genetic background, as the number of laid eggs was comparable between *C507-Gal4^ts^/+* females and *C507-Gal4^ts^>UAS-rpr+hid* females without induced remodeling of MTs (Fig. 4B). Together, these results indicate that RSC-mediated remodeling of adult MTs modestly reduces female fecundity.

To further assess the differences between the remodeled renal tubules and uninjured renal tubules, we compared the transcriptomes of the SCZ before and after remodeling using mRNA sequencing (mRNA-seq). Principal components analysis and hierarchical clustering based on global gene expression showed that control and remodeled lower tubules express similar transcriptomes (Fig. 4C and fig. S6A), suggesting that they support very similar physiological functions. Nevertheless, the transcriptomes were not identical. Six hundred fifty-two genes were differentially expressed with fold change > 2 and *P*_adj_ < 0.05 between remodeled and control MTs using DESeq2 (*17*). Among these genes, 360 genes were up-regulated, whereas 292 genes were down-regulated in remodeled MTs compared to control. We subsequently performed Gene Ontology (GO) enrichment analysis using Metascape (*18*). ECM-receptor interaction (dmel04512) stood out as the top enriched GO term among the up-regulated genes. ECM genes including *vkg*, *Col4a1*, *LanB1*, *LanA*, *troll*, *wb*, *tig*, *tsp*, and *ppn* were all up-regulated after remodeling, agreeing with our previous observation of a thickened ECM following remodeling (fig. S6, B and C). These changes may be needed to maintain mechanical stability in light of the increased tubule diameter. In addition, a group of genes involved in transmembrane transport including sodium ion transport and potassium ion transport were up-regulated, making the “GO:0055085 transmembrane transport” the second most enriched GO term among up-regulated genes (fig. S6, B and C).

Given that the expression of transmembrane transport genes is already elevated in remodeled MTs under normal condition, we reasoned that these tubules might be more sensitive to salt stress. Consequently, we modified a previously reported salt stress assay to examine the salt tolerance capacity of flies bearing wild-type MTs or remodeled MTs (*19*). The survival of flies bearing wild-type MTs or remodeled MTs was comparable when fed a 5% sucrose-only diet. In contrast, flies bearing remodeled MTs died substantially faster than control animals when subjected to a high salt stress diet containing 250 mM NaCl mixed with 5% sucrose (Fig. 4D). These results suggest that remodeling of adult MTs compromises their salt tolerance, possibly because an increased tubule diameter changes the surface-to-volume ratio in an unfavorable manner for processes such as salt resistance that are highly dependent on surface-localized transporters.

## DISCUSSION

Previous studies of stem cells that maintain tissues with active cell turnover suggest that they function to maintain tissue homeostasis in the face of cell loss, environmentally mediated damage, and fluctuating resources [reviewed in (*1*–*3*, *20*–*22*)]. By adjusting rates of cell production and proportions of different downstream cell types and engaging in competition with genetically distinct cells for retention in a niche, the essential structure and function of many tissues can be maintained by homeostatic stem cells throughout life. However, our experiments show that RSCs represent a different class of stem cells. RSC-mediated repair of adult *Drosophila* lower tubules does not restore tissue morphology and homeostasis but mediates an irreversible tissue makeover, which takes place only once during adulthood. If the effects were uniformly positive, then RSCs would likely resemble other developmental progenitors and carry out beneficial remodeling before adulthood or during major tissue regeneration. However, we found that the RSC-mediated makeover can exert either positive and negative fitness effects depending on circumstances beyond organismal control.

RSCs appear to be developmental progenitors that evolution has transformed into stem cells that persist into adulthood. This is similar to the proposed origin of female germline stem cells from primordial germ cells that become stabilized in a niche rather than further developing into germ cells (*23*). However, RSCs are progenitors stabilized by becoming quiescent because this maximizes the ability to control when their morphological remodeling takes place. In many instances, this would occur after reproduction has largely taken place but before kidney stones have had time to form. We suggest that this class of stem cells be called “makeover stem cells” and suggest that they are fairly common. However, makeover stem cells are much harder to identify than homeostatic stem cells because they will remain quiescent under most circumstances, resembling differentiated tissue cells, such as RSCs. Only under the appropriate conditions will they manifest their latent stem cell character and endogenous developmental program.

Mammalian kidneys may have makeover stem cells that are similar to RSCs. Our studies highlight the notable similarities in the process of renal stone formation between *Drosophila* and mammals (*13*, *14*). Loss of *XDH* generates frequent xanthine stones in both *Drosophila* and human excretory systems. Renal stones often first strike the *Drosophila* lower tubule, which acts to concentrate the tubular fluid and controls fluid flow via constriction of encircling ureter muscles. The mammalian counterparts of the *Drosophila* lower tubule with analogous functions are the renal papillae and renal pelvis (*24*). Randall’s plaques that are believed to act as nidi for urinary stone formation are predominantly attached to renal papillae protruding into the renal pelvis (*25*), similar to the MT brush border. Quiescent kidney papillary label-retaining cells have been reported to be able to regenerate medullar tubules upon severe kidney injury in mice (*26*, *27*). It would be interesting to learn whether medullar tubule regeneration alters tissue morphology and increases stone resistance in mammals. Deepening our understanding of the cellular and molecular mechanisms underlying renal stone formation and cellular responses via makeover stem cells such as RSCs and possible mammalian counterparts will advance our ability to prevent and treat kidney stones, a growing medical and economic burden globally (*12*).

## MATERIALS AND METHODS

### *Drosophila* stocks and husbandry

*Drosophila* stocks are reared on standard cornmeal molasses yeast food and maintained at room temperature (23° to 25°C), unless otherwise specified. A dash of dry yeast is routinely sprinkled on the surface of cornmeal molasses yeast food before use, unless otherwise specified. The following stocks were used in this study: *ry^506^* [Bloomington Drosophila Stock Center (BDSC), #4405], *OregonR* (BDSC, #25211), *C507-Gal4* (BDSC, #30840), *tub-Gal80^ts^* (BDSC, #7019), *UAS-RFP* (BDSC, #32222), *esg-Gal4;tub-Gal80^ts^,UAS-GFP* (*28*), and *UAS-rpr,UAS-hid* (*29*).

### Immunostaining and microscopy

MTs left attached to the gut were dissected in Grace’s insect medium or 1× phosphate-buffered saline (PBS). Samples were then fixed in 4% paraformaldehyde in PBS on a nutator for 20 min. After washing three times with PBT (1× PBS with 0.1% Triton X-100) for 5 min each, samples were blocked with 5% normal goat serum in PBT for 1 hour followed by incubating with primary antibody at 4°C overnight. Samples were washed three times with PBT and then incubated in secondary antibody for 2 to 3 hours at room temperature or 4°C overnight. After three 5-min washes with PBT, samples were counterstained with 4′,6-diamidino-2-phenylindole (DAPI) (100 ng/ml) (and 1:1000 fluorophore-conjugated phalloidin if needed) for 5 min at room temperature. Samples were then washed twice with PBT for 5 min each and mounted in 50% glycerol. The antibodies used in this study are the following: mouse anti–lamin C [1:10; Developmental Studies Hybridoma Bank (DSHB), catalog no. LC28.26], mouse anti-Coracle (1:50; DSHB, catalog no. C566.9), mouse anti-Armadillo (1:5; DSHB, catalog no. N27A1), mouse anti-Cut (1:10; DSHB, catalog no. 2B10), Alexa Fluor 568 goat anti-mouse (1:300; Thermo Fisher Scientific, catalog no. A11004), and Alexa Fluor 488 goat anti-mouse (1:300; Thermo Fisher Scientific, catalog no. A11001). Fluorescence images were taken with a Leica TCS SP8 confocal microscope.

### Induced remodeling of the SCZ

We induced remodeling of the SCZ using genetic ablation of preexisting PCs as previously described (*5*). Briefly, *tub-Gal80^ts^;C507-Gal4, UAS-RFP* (referred to as *C507-Gal4^ts^* for brevity) female flies were crossed to *UAS-rpr,UAS-hid* animals to produce *C507-Gal4 ^ts^>UAS-rpr,hid* flies at 18°C. Three- to 5-day-old *C507-Gal4^ts^>UAS-rpr+hid* flies were first shifted to 29°C for 7 days (“injured”) and then shifted back to 18°C for at least 14 days to allow completion of remodeling. *C507-Gal4^ts^* female flies were crossed to *OregonR* males to produce *C507-Gal4^ts^/+* animals, which were subjected to the same temperature regimen as the experimental flies and were used as control.

### Total volume of nuclei in the SCZ

The wild-type SCZ was marked by *C507-Gal4>RFP*. The regenerated SCZ can be easily discerned as the replacement PCs are much smaller compared to those in the upper tubules. z-stack images were acquired using a Leica TCS SP8 confocal microscope with a 20× objective lens (numerical aperture = 0.75). The SCZ was selected to make three-dimensional surface reconstruction of nuclei based on DAPI staining. The volume of surface-masked SCZ was calculated by IMARIS v9.2.1 (Bitplane, RRID: SCR_007370).

### Flies for RNA-seq

C507-Gal4^ts^ animals were crossed to *OregonR* or *UAS-rpr,UAS-hid* animals at 18°C. Three- to 5-day-old *C507-Gal4^ts^/+* females and *C507-Gal4^ts^>UAS-rpr+hid* females were selected from above crosses and were subjected to temperature shift paradigm. After shifting to 29°C for 7 days, half of the *C507-Gal4^ts^/+* females and *C507-Gal4^ts^>UAS-rpr+hid* females were dissected to collect the ureters and lower tubules. The samples were referred to as “Ctrl (29°C)” and injured, respectively. The rest of *C507-Gal4^ts^/+* females and *C507-Gal4^ts^>UAS-rpr+hid* females were shifted back to 18°C for 21 days before dissecting the ureters and lower tubules. These samples were referred to as “Ctrl (29°C–18°C)” and “remodeled,” respectively. About 100 flies were dissected in nuclease-free PBS on ice for each replicate. The samples were stored in 500 μl of TRIzol (Invitrogen, 15596026) and stored at −80°C until all samples were ready for RNA extraction.

### mRNA sequencing

The RNAs were then extracted following the manufacturer’s instructions. cDNA libraries were processed as previously described (*5*). Seventy-five–base pair single-end reads were aligned to the *D. melanogaster* genome (dm6) using HISAT2 2.1.0 (*30*). Read counts per gene were calculated using htseq-count (*31*). Differentially expressed genes were identified using DESeq2 (*17*).

### Electron microscopy

Samples from flies with desired genotypes were dissected and processed as previously described (*5*).

### Measurement of brush border and ECM

The brush border length and ECM thickness were measured using electron microscopy (EM) images at ×4000 or ×20,000 magnification from at least eight different tubules of indicated genotypes, respectively. For each EM image, five to eight subregions were randomly selected to be measured in Fiji (*32*).

### Egg laying

For egg laying assays, females of appropriate genotypes were subjected to the temperature shift to induce remodeling of MTs as described above. *OregonR* males were provided to mate with females at 1:1 ratio throughout the temperature shift. After 14 days at 18°C, males were replaced with young *OregonR* males. Twenty pairs of males and females of each genotype were subsequently divided into four groups and transferred to four separate bottles containing molasses plate with wet yeast at 20°C. The molasses plates were changed every 24 hours, and the eggs laid on the plates were scored every 24 hours for six consecutive days.

### Survival under high salt stress condition

For salt feeding, a previous reported assay was modified by adding 1% melted agarose to the liquid diet to make solid food (*19*). Control flies and flies with remodeled MTs were switched from normal food to the agarose-based diets containing 5% sucrose only (as control) or 5% sucrose with 250 mM NaCl mixed at 18°C. Flies were examined for survival daily and transferred to fresh food every other day.

### Allopurinol injection

Flies with appropriate genotypes were injected with about 100 nl of injection solution [3 mM allopurinol mixed with Blue No. 1 (10 mg/ml) in 1× PBS] and then transferred to regular corn meal food containing 3 mM allopurinol. A dash of dry yeast was sprinkled on food surface before use. Flies were injected for four consecutive days with an interval of 24 hours. Survival of the injections on all days was more than 90%.

### Quantification of stone area

*Drosophila* MTs left attached to the gut were dissected in Grace’s insect medium or 1× PBS. MTs were then imaged with a Nikon SMZ1500 stereomicroscope equipped with HR Plan Apo 1× WD 54 objective, HR Plan Apo 1× WD 54 objective, and Infinity 3 Lumenera camera within 30 min after dissection. Stones were manually outlined using the freehand selection tool in Fiji, and the area was acquired using Fiji (*32*).

### Statistical analysis

Student’s *t* test was used to determine the statistical significance in most cases except for the laid egg comparison and survival rate comparison, for which one-way repeated measures analysis of variance (ANOVA) and log-rank test were used, respectively. ns, *P* > 0.05; *P < 0.05, **P < 0.01, and ***P < 0.001.

## Notes

View/request a protocol for this paper from *Bio-protocol*.
