# Supplementary material for: Drosophila renal stem cells enhance fitness by delayed remodeling of adult Malpighian tubules
Source: Sci Adv. 2022 May 20;8(20):eabn7436. doi: 10.1126/sciadv.abn7436 (PMC9122315; doi:10.1126/sciadv.abn7436)
Supplement: Supplementary file 1 — Figs. S1 to S6 [file sciadv.abn7436_sm.pdf]

Science Advances
AAAS

Supplementary Materials for

Drosophila renal stem cells enhance fitness by delayed remodeling of adult Malpighian tubules

Chenhui Wang and Allan C. Spradling*

*Corresponding author. Email: spradling@carnegiescience.edu

Published 20 May 2022, *Sci. Adv.* **8**, eabn7436 (2022)
DOI: 10.1126/sciadv.abn7436

This PDF file includes:

Figs. S1 to S6

Supplementary Figures

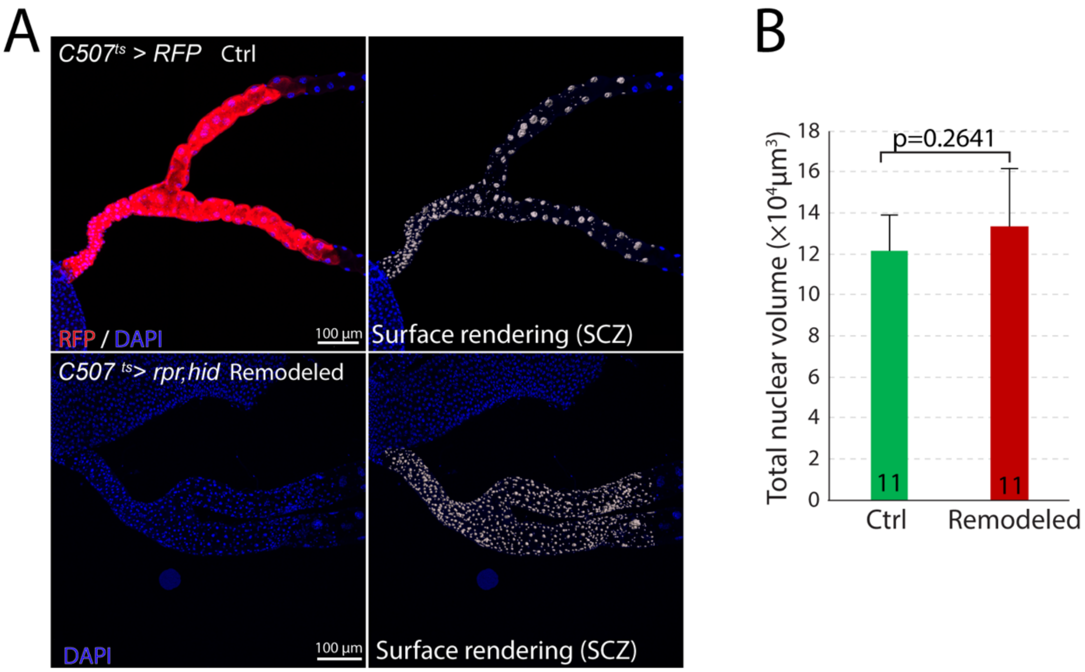

Fig. S1. The total DNA content of the SCZ can be restored after remodeling. (A) Representative micrographs of the SCZ from control and regenerated MTs. The 3D surface rendering of the nuclei in the SCZ was based on DAPI staining using IMARIS software. **(B)** The overall volume of nuclei is comparable between control and regenerated SCZ.

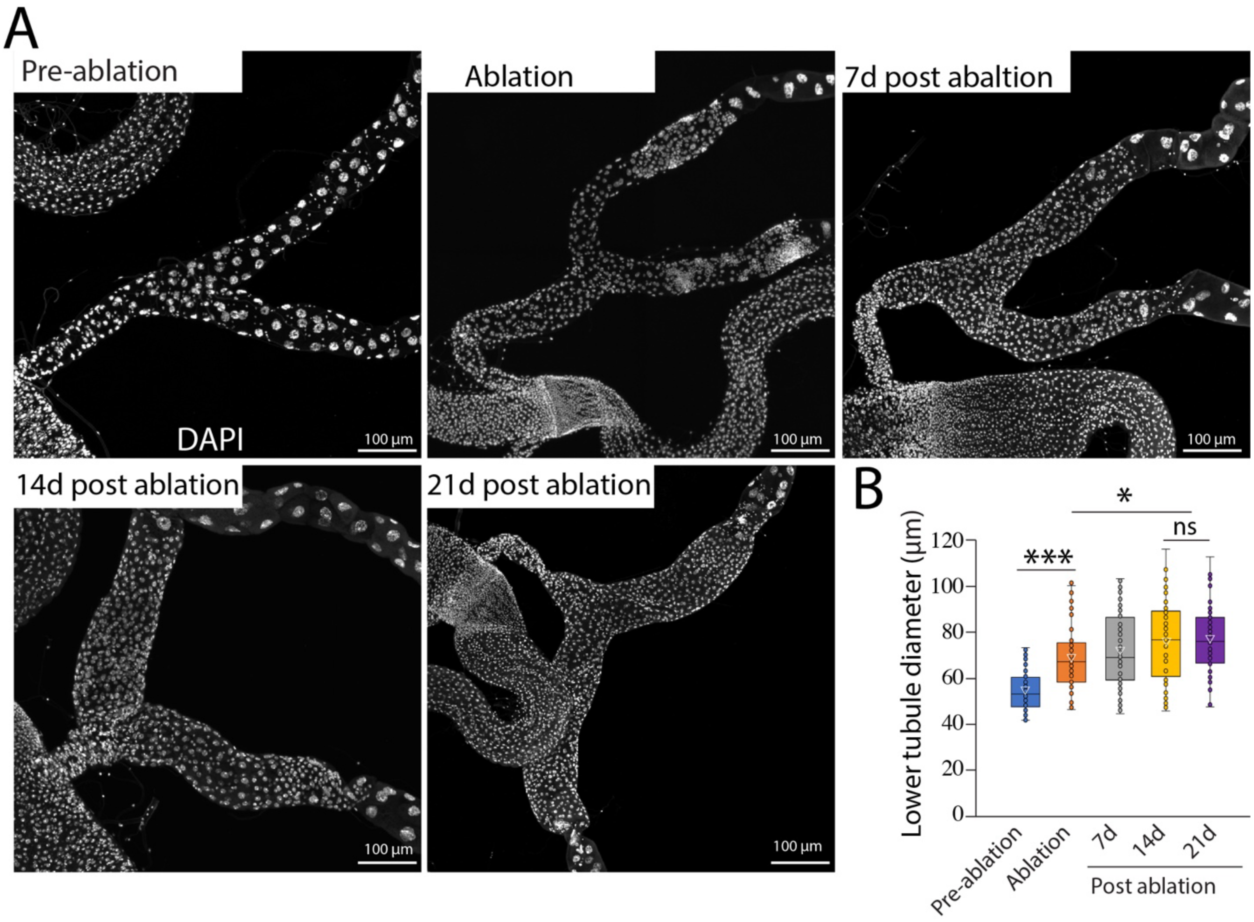

Fig. S2. Remodeling increases the diameter of lower tubules. **(A)** Micrographs showing the lower tubules of *C507-Gal4^{ts} >UAS-rpr+hid* flies over the course of remodeling. **(B)** Box and whisker plot for the quantification of lower tubule diameter. The white triangle indicates the mean value, whereas the line inside the box indicates median value. The two lines constitute the top and bottom of the box are the 25th and 75th percentiles respectively. *** denotes two-tailed Student's t test p<0.001, * p <0.05, ns: not significant (p>0.05). Scale bars as indicated.

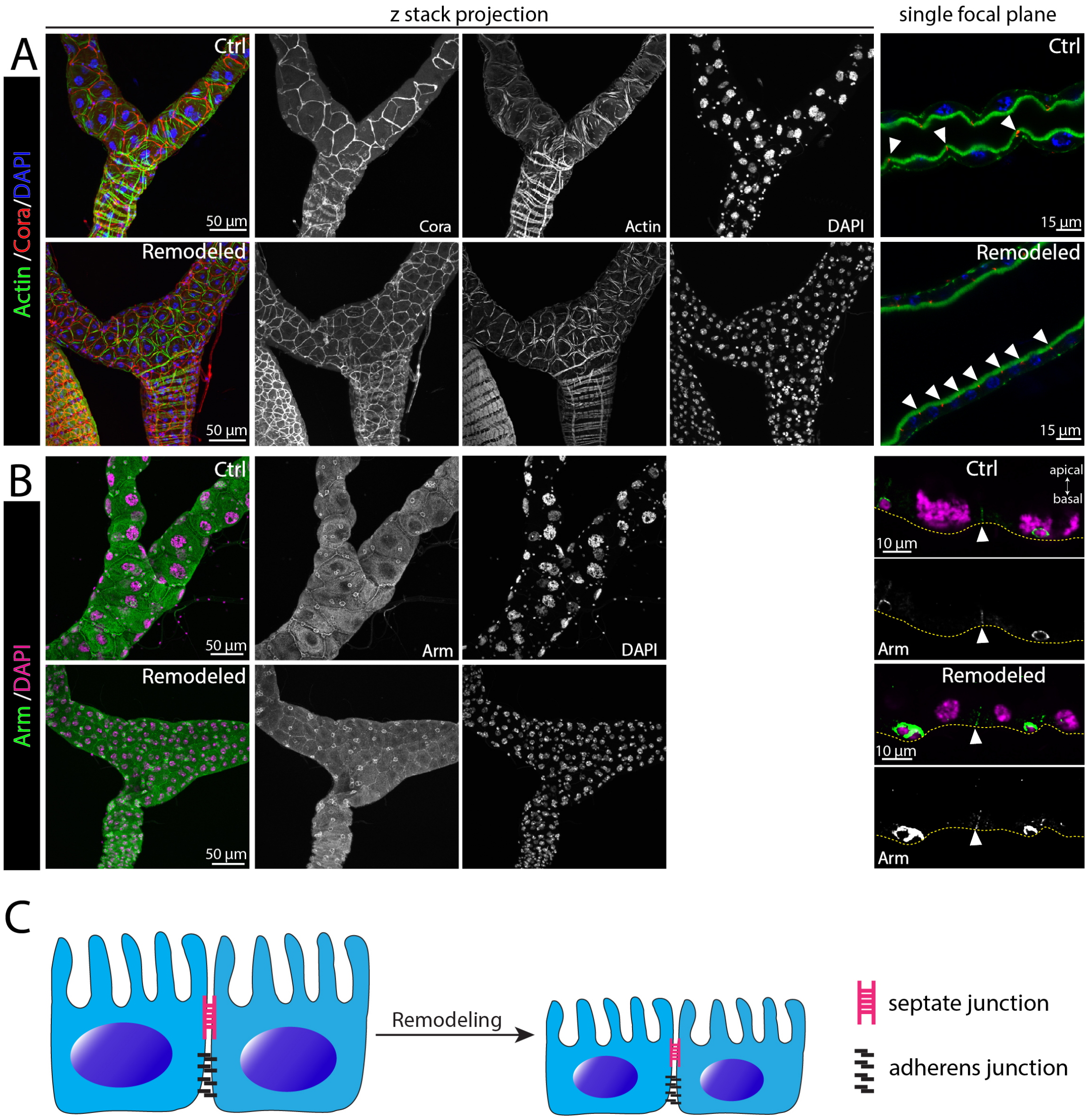

Fig. S3. The barrier integrity of Malpighian tubules can be restored following repair. (A) Counterstaining of F-actin cytoskeleton and septate junction component Cora in control and regenerated SCZ. Note the apicolateral localization of Cora (denoted by white triangles) in preexisting principal cells as well as regenerated principal cells. **(B)** Staining of the adherens junction protein Arm in the wild type and regenerated SCZ. Note the basolateral localization of Arm (denoted by white triangles) in preexisting principal cells as well as regenerated principal

cells. **(C)** A drawing depicting the morphological remodeling of adult lower tubules upon regeneration.

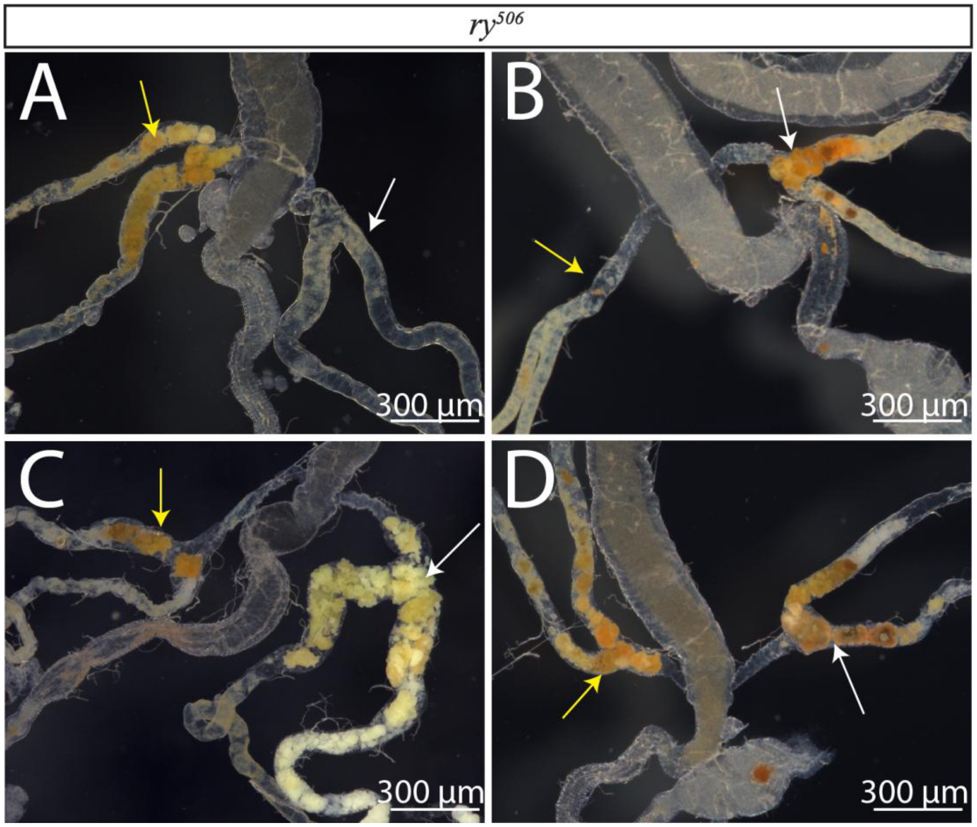

Fig. S4. Obstructing xanthine stones caused by loss of *ry*. (A-D) Micrographs showing xanthine stones in anterior pair (denoted by white arrows) and posterior pair (denoted by yellow arrows) of MTs from *ry* mutants. Xanthine stone formation in aMTs and in pMTs are seemingly independent events.

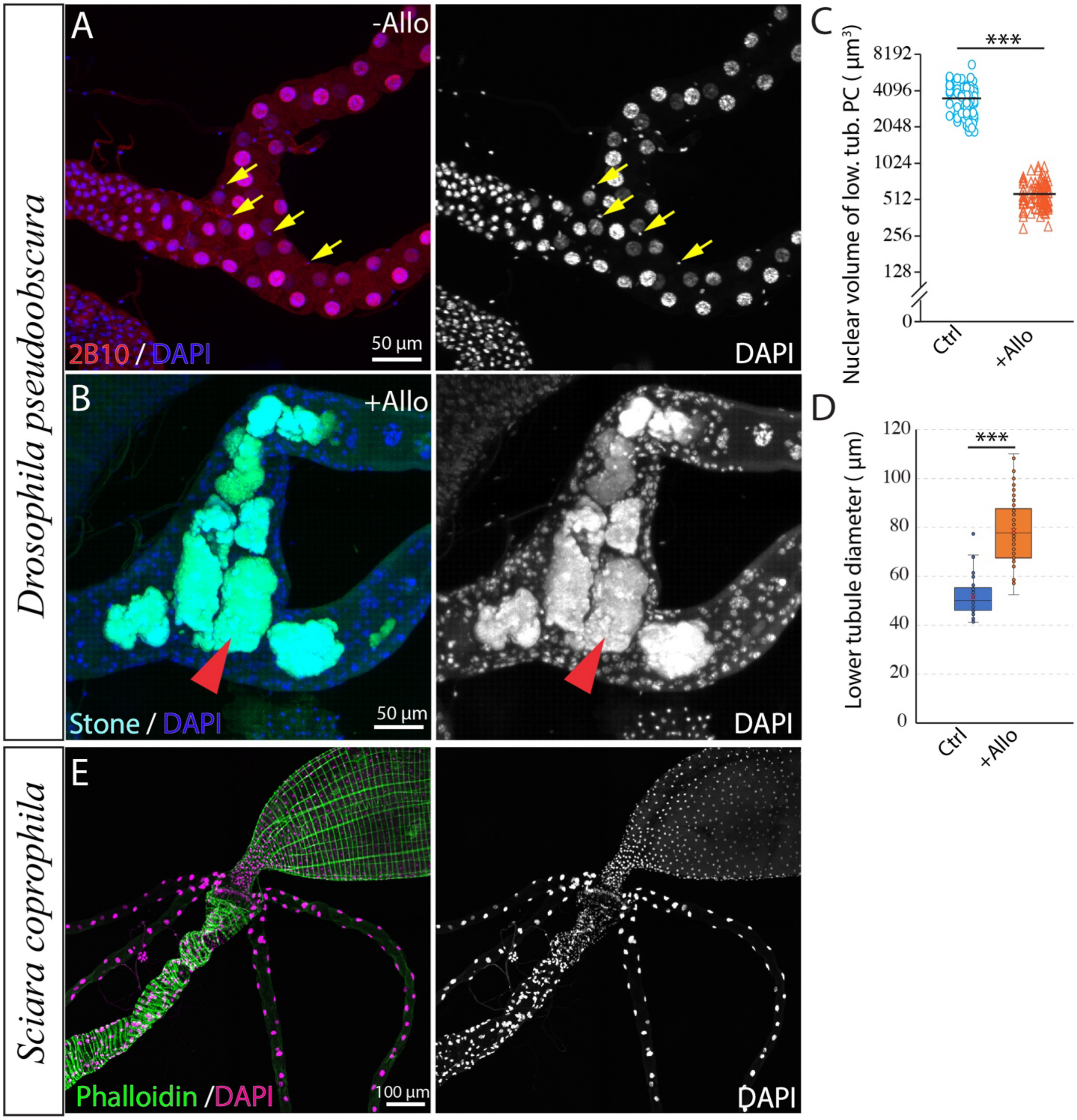

Fig. S5. Conserved mode of RSC-mediated repair of adult MTs between *Drosophila melanogaster* and *Drosophila pseudoobscura*. (A) Immunofluorescence micrograph of the ureter and lower tubules of an adult female wildtype *Drosophila pseudoobscura* stained with 2B10 antibody (anti-Cut) under normal condition. Yellow arrows denote diploid cells that are presumably renal stem cells. **(B)** Representative image of the ureter and lower tubules of

Drosophila pseudoobscura carrying Allopurinol-induced xanthine stones (red triangles). Note the supernumerary cells adjacent to xanthine stones. **(C)** Quantification of nuclear volume of preexisting PCs and replacement PCs in the SCZ from animals without or with Allopurinol treatment, respectively. (**D**)Quantification of lower tubule diameter in *D.pseudoobscura* without or with Allopurinol treatment, respectively. **(E)** Representative image of the ureter and lower tubules of an adult female wildtype *Sciara coprophila* stained with phalloidin. Note the absence of diploid cell population in the tubule epithelium. Bar denotes the average value. *** denotes $p<0.001$ with Student's t test.

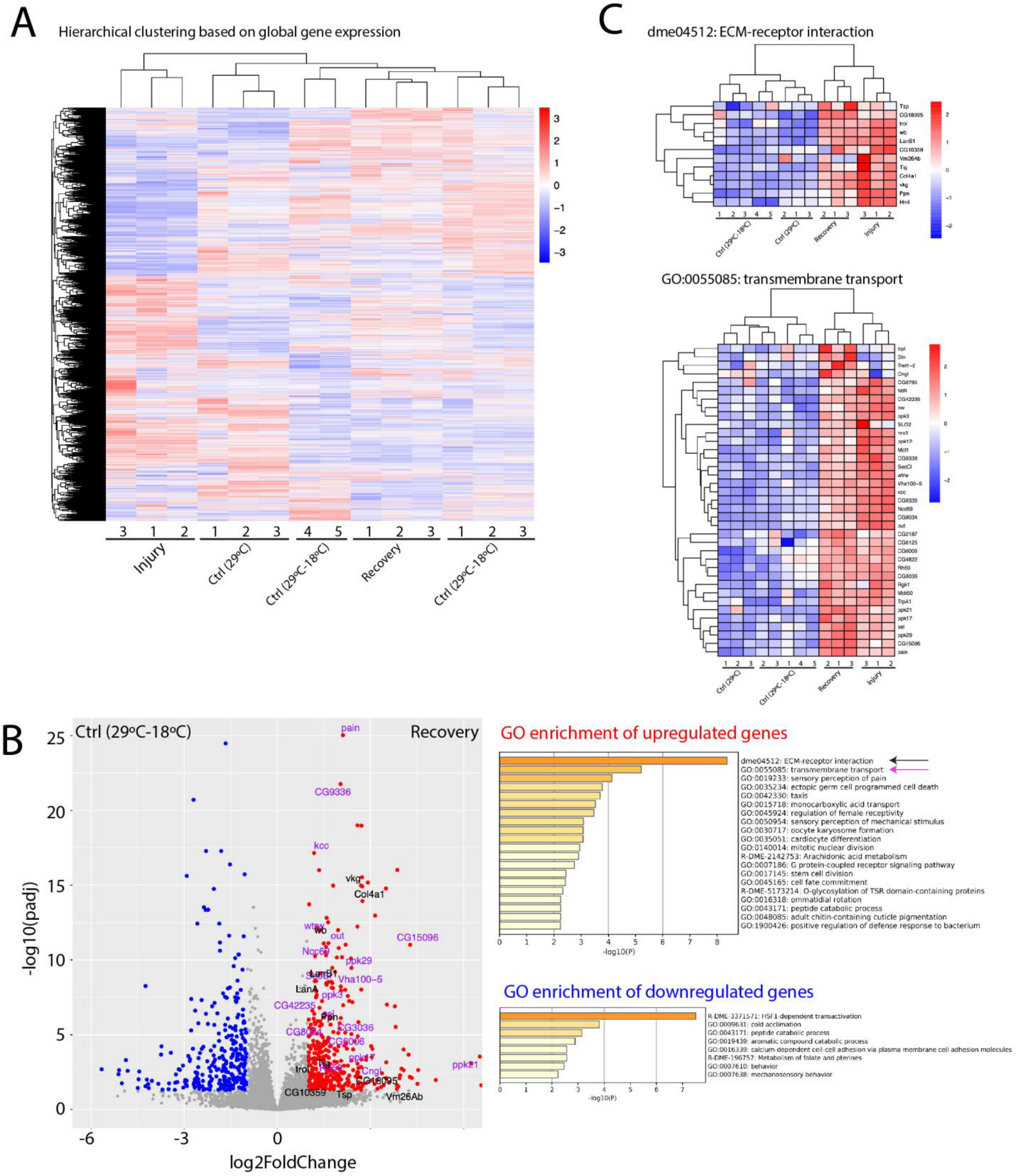

Fig. S6. Transcriptome comparison between the SCZ of remodeled MTs and that of control MTs. **(A)** Heatmap showing the hierarchical clustering of samples based on global gene expression. **(B)** Left: Volcano plot showing the differentially expressed genes between the

transcriptomes of Remodeled and Ctrl (29°C - 18°C) groups. Differentially expressed genes (padj<0.05, FoldChange >2) were identified using DESeq2. Upregulated genes and downregulated genes after remodeling are shown in red and blue dots, respectively. Right: GO enrichment of upregulated and downregulated genes respectively. GO enrichment analysis was performed using Metascape (*18*). **(C)** Heatmap showing the genes involved in ECM-receptor interaction (upper) and transmembrane transport (lower) that were upregulated in MTs after remodeling compared with control, respectively.
